# Silent Changes in Sleep Quality Following Mandibular Setback Surgery in Patients with Skeletal Class III Malocclusion: A Prospective Study

**DOI:** 10.1038/s41598-019-46166-z

**Published:** 2019-07-05

**Authors:** Sung Woon On, Hyun Jun Kim, Dong Hyeon Cho, Yeo Rae Moon, Seung Il Song

**Affiliations:** 1 Division of Oral and Maxillofacial Surgery, Department of Dentistry, Dongtan Sacred Heart Hospital, Hallym University College of Medicine, Hwaseong, Republic of Korea; 2 Department of Otolaryngology, Ajou University School of Medicine, Suwon, Republic of Korea; 3 Department of Oral and Maxillofacial surgery, Ajou University Dental Hospital, Suwon, Republic of Korea; 4 Office of Biostatics, Ajou Research Institute for Innovation Medicine, Ajou University Medical Center, Suwon, Republic of Korea

**Keywords:** Malocclusion, Craniofacial orthodontics

## Abstract

Mandibular setback surgery (MSS) for skeletal class III patients can result in a relative reduction of pharyngeal airway space (PAS). Consequently, there is a possibility of the decline of sleep quality after surgery. We investigated changes in sleep quality measured by overnight polysomnography (PSG) and the three-dimensional (3D) volumes of PAS following MSS with or without Le Fort I osteotomy (LF I) in class III patients (N = 53). Overnight PSG and cone beam computed tomography were conducted at preoperative stage (T0) and postoperative 3 months (T1). Measurements of PAS volumes were performed, and the subjective symptoms of sleep were evaluated by self-questionnaires. There were significant increases in respiratory disturbance index (RDI) and total respiratory effort-related arousal (RERA) index during T0-T1. The 3D volumes of PAS showed significant decreases in the oropharyngeal airway, hypopharyngeal airway, and total airway spaces. No significant changes were observed in subjective symptoms of sleep. MSS with or without LF I for class III patients could worsen sleep quality by increasing sleep parameters such as the RDI and RERA in PSG, and reduce volumes of PAS at postoperative 3 months. Although subjective symptoms may not show significant changes, objective sleep quality in PSG might decrease after MSS.

## Introduction

For the past two decades, researchers have debated whether mandibular setback surgery (MSS) for skeletal class III patients can reduce the upper airway dimension, and consequently provoke sleep-related breathing disorders such as obstructive sleep apnea (OSA). Currently, it is widely accepted that a relative reduction of the pharyngeal airway space (PAS) could be resulted from mandibular setback. Although some papers have reported patients who were diagnosed with mild OSA after MSS^1,2^, there is no evidence for postoperative OSA^3–5^. However, many authors have recommended simultaneous double jaw surgery consisting of maxillary advancement and mandibular setback for severe class III patients not only to reduce the amount of mandibular setback, but also to lower the risk of pharyngeal airway compromise because of the possibility of postoperative OSA^6–9^.

Although a vast majority of studies have focused on changes in the upper airway or the possibility of OSA after orthognathic surgery, few studies have addressed changes in the sleep parameters in overnight polysomnography (PSG) following MSS. The sleep parameters in PSG consist of numerous variables related to respiratory function and sleep quality. In particular, some parameters including respiratory effort-related arousal (RERA) and snoring time can indicate the quality of sleep, and the exacerbation of these parameters might cause significant symptoms^10^. However, most studies using overnight PSG have concentrated on changes in the apnea-hypopnea index (AHI) or lowest oxygen desaturation after mandibular setback, and the results of PSG examination have not been described in detail. In addition, there is a need to find out the correlations between changes in sleep parameters in PSG and volumetric changes in the pharyngeal airway dimension using computed tomography. Despite the reduction of PAS caused by mandibular setback, the occurrence of postoperative OSA is inconsistent with changes in the upper airway dimension. Therefore, it is logical to investigate the correlations between the sleep parameters in PSG and three-dimensional (3D) changes in upper airway space for identifying the unbeknown factors involved in OSA.

The aims of the present study were: (1) to investigate changes in the sleep parameters of PSG and the 3D volume of PAS following MSS with or without maxillary movement in skeletal class III patients; (2) to identify possible correlations among the sleep parameters in PSG, volumetric changes in PAS, subjective symptoms of patients, and other demographic data such as the amounts of surgical movements of the mandible; and (3) to elucidate the factors affecting the occurrence of OSA after MSS.

## Results

### Changes in the sleep parameters in PSG, 3D Volumes of PAS and subjective symptoms of sleep based on self-questionnaires between the preoperative stage (T0) and postoperative 3 months (T1)

Among the sleep parameters in PSG during T0–T1, there were significant increases in the respiratory disturbance index (RDI), and total RERA index (both *P* < 0.001; Table 1). On the other hand, body mass index (BMI) decreased significantly during T0–T1 (*P* < 0.001; Table 1).

**Table 1:** Changes in variables of PSG, and CBCT data between the T0 and T1 stages.

Variables		N	T0			T1			Wilcoxon Z	Adjusted *P* Value^*^
			Median	Min.	Max.	Median	Min.	Max.		
PSG data	AHI (number/h)	50	1.15	0.00	12.40	1.10	0.00	28.70	−2.396	0.391
	RDI (number/h)^**^	50	4.10	0.20	21.20	6.60	0.10	41.20	−3.482	<0.001
	BMI (kg/m^2^)^**^	50	22.65	15.30	33.30	22.45	14.90	33.30	−4.041	<0.001
	Total sleep time (minutes)	50	401.50	205.00	462.30	409.75	167.00	475.90	−1.168	1.000
	Sleep efficiency (%)	50	86.75	40.70	97.60	91.15	33.60	98.20	−2.018	1.000
	Wake after sleep onset (minutes)	50	50.50	7.40	280.50	30.25	5.00	322.50	−2.528	1.000
	Number of awakening	50	26.00	4.00	68.00	26.00	10.00	61.00	−1.626	1.000
	N1 stage percentage (%)	50	9.45	2.40	30.40	9.30	1.20	29.10	−1.651	1.000
	N2 stage percentage (%)	50	56.35	33.10	70.10	57.40	36.50	71.50	−0.275	1.000
	N3 stage percentage (%)	50	14.50	0.00	32.70	13.70	0.00	27.10	−1.646	1.000
	REM stage percentage (%)	50	17.55	3.80	27.60	19.60	9.30	32.90	−2.636	0.184
	Total arousal index (number/h)	50	14.90	5.30	30.10	15.30	6.70	34.30	−0.285	1.000
	Total RERA index (number/h)^**^	50	2.55	0.00	16.20	4.70	0.00	16.60	−3.498	<0.001
	Relative snoring time (%)	50	3.40	0.00	52.80	8.70	0.00	71.00	−2.578	0.230
	Mean oxygen saturation (%)	50	97.20	94.80	98.20	97.60	95.40	98.60	−2.721	0.161
	Lowest oxygen saturation (%)	50	93.00	85.00	96.00	94.00	76.00	97.00	−0.103	1.000
	Average oxygen saturation during wake (%)	50	97.70	95.40	98.60	97.85	96.20	98.70	−2.065	0.897
	Average oxygen saturation during NREM (%)	50	97.20	94.60	98.10	97.60	95.30	98.60	−2.848	0.368
	Average oxygen saturation during REM (%)	50	97.35	95.60	98.60	97.80	94.70	98.60	−2.381	1.000
CBCT data	Volume of total pharyngeal airway space (cc)^**^	50	23.85	9.50	61.30	20.03	6.35	42.95	−4.148	<0.001
	Volume of nasopharyngeal airway space (cc)	50	5.78	1.85	12.35	5.25	1.45	12.20	−2.472	0.299
	Volume of oropharyngeal airway space (cc)^**^	50	7.88	2.75	23.30	6.80	0.90	14.25	−3.862	<0.001
	Volume of hypopharyngeal airway space (cc)^**^	50	9.18	1.80	30.95	8.53	2.35	25.35	−3.543	<0.001

^*^Statistical analysis was carried out using Wilcoxon-signed rank test (before vs. after surgery), and Bonferroni adjustment was applied for multiple comparison correction. ^**^Variables examined by performing one-tailed test (before vs. after surgery).

In terms of the 3D volumes of PAS, there were significant decreases in the oropharyngeal airway, hypopharyngeal airway, and total airway spaces (all *P* < 0.001; Table 1).

Regarding subjective symptoms, neither the Epworth Sleepiness Scale (ESS) nor the Pittsburgh Sleep Quality Index (PSQI) scores showed significant changes (Table 2).

**Table 2:** Changes in scores of self-questionnaires between the T0 and T1 stages.

Variable	N	T0			T1			Wilcoxon Z	Adjusted *P* Value^*^
		Median	Min.	Max.	Median	Min.	Max.		
ESS total score	36	7.00	0.00	14.00	8.00	1.00	15.00	−1.417	0.312
PSQI total score	36	5.00	1.00	11.00	5.00	2.00	10.00	−0.176	1.000

^*^Statistical analysis was carried out using Wilcoxon-signed rank test (before vs. after surgery), and Bonferroni adjustment was applied for multiple comparison correction.

### Correlations among the sleep parameters in PSG, volumetric changes in PAS, subjective symptoms of patients, and other demographic data

**Figure 1:**
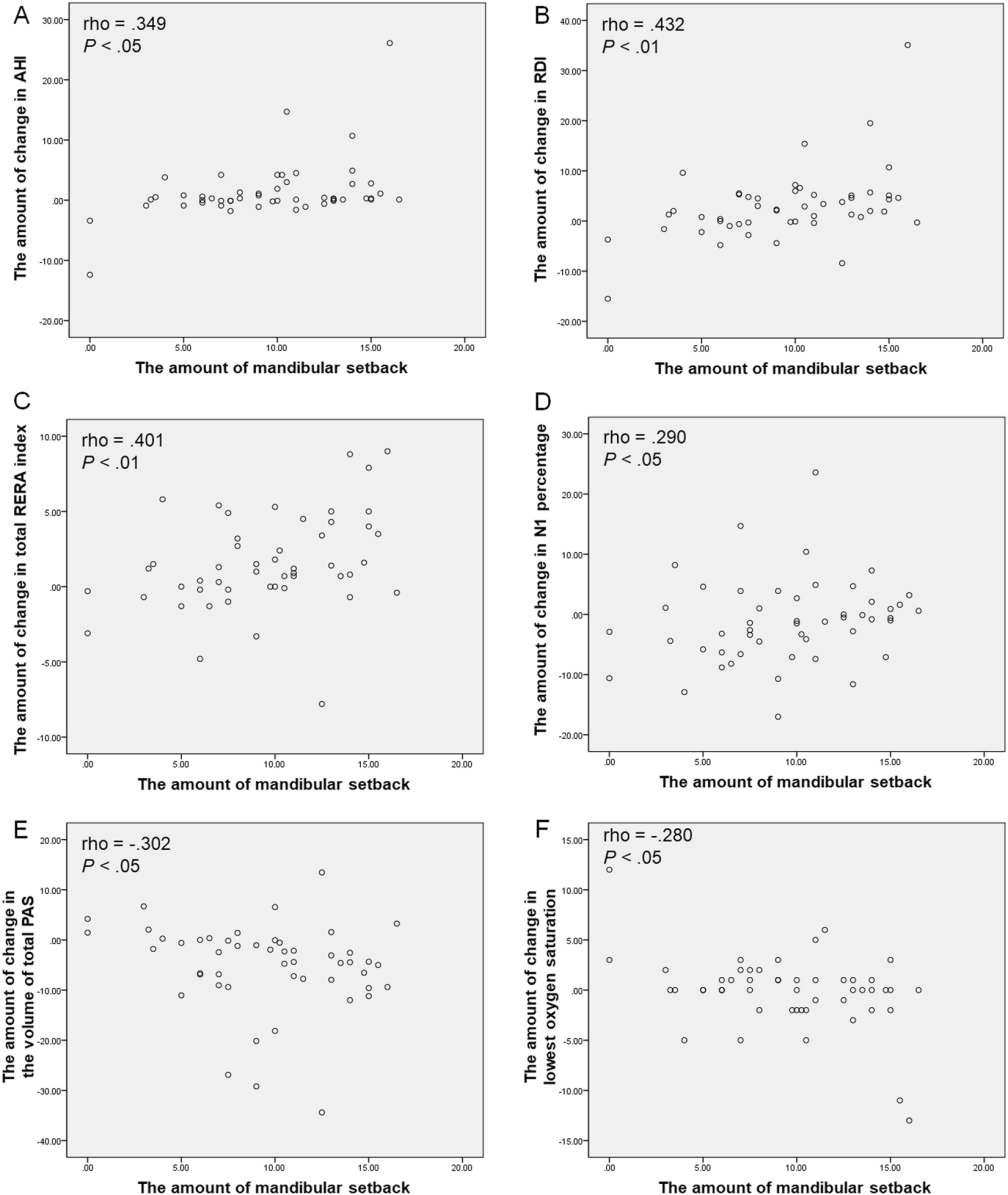
Scatter plots showing positive correlations between the amounts of mandibular setback and the changes in other variables. The changes in the AHI **(A)**, RDI **(B)**, total RERA index **(C)**, and N1 percentage **(D)** showed positive correlation with the amounts of mandibular setback. On the other hand, changes in the volume of total PAS **(E)**, and the lowest oxygen saturation **(F)** showed negative correlation with the amounts of mandibular setback.

Significant correlations were found between the sleep parameters in PSG and the amount of mandibular setback. The changes in the AHI, RDI, total RERA index, and N1 percentage correlated positively with the amount of mandibular setback (rho, 0.349, *P* < 0.05; rho, 0.432, *P* < 0.01; rho, 0.401, *P* < 0.01; rho, 0.290, *P* < 0.05; respectively) (Fig. 1), whereas the changes in lowest oxygen saturation, and total airway space correlated negatively with the amounts of mandibular setback (rho, −0.280, *P* < 0.05; rho, −0.302, *P* < 0.05; respectively) (Fig. 1).

**Figure 2:**
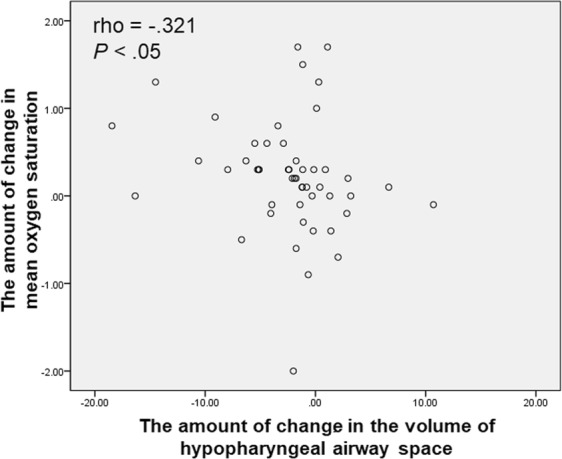
Scatter plot showing the correlation between the changes in mean oxygen saturation and the volume of hypopharyngeal airway space.

Regarding correlations between the parameters in PSG and the volume changes in PAS, changes in mean oxygen saturation only showed significant negative correlation with changes in the volumes of hypopharyngeal airway space (rho, −0.321, *P* < 0.05) (Fig. 2).

**Figure 3:**
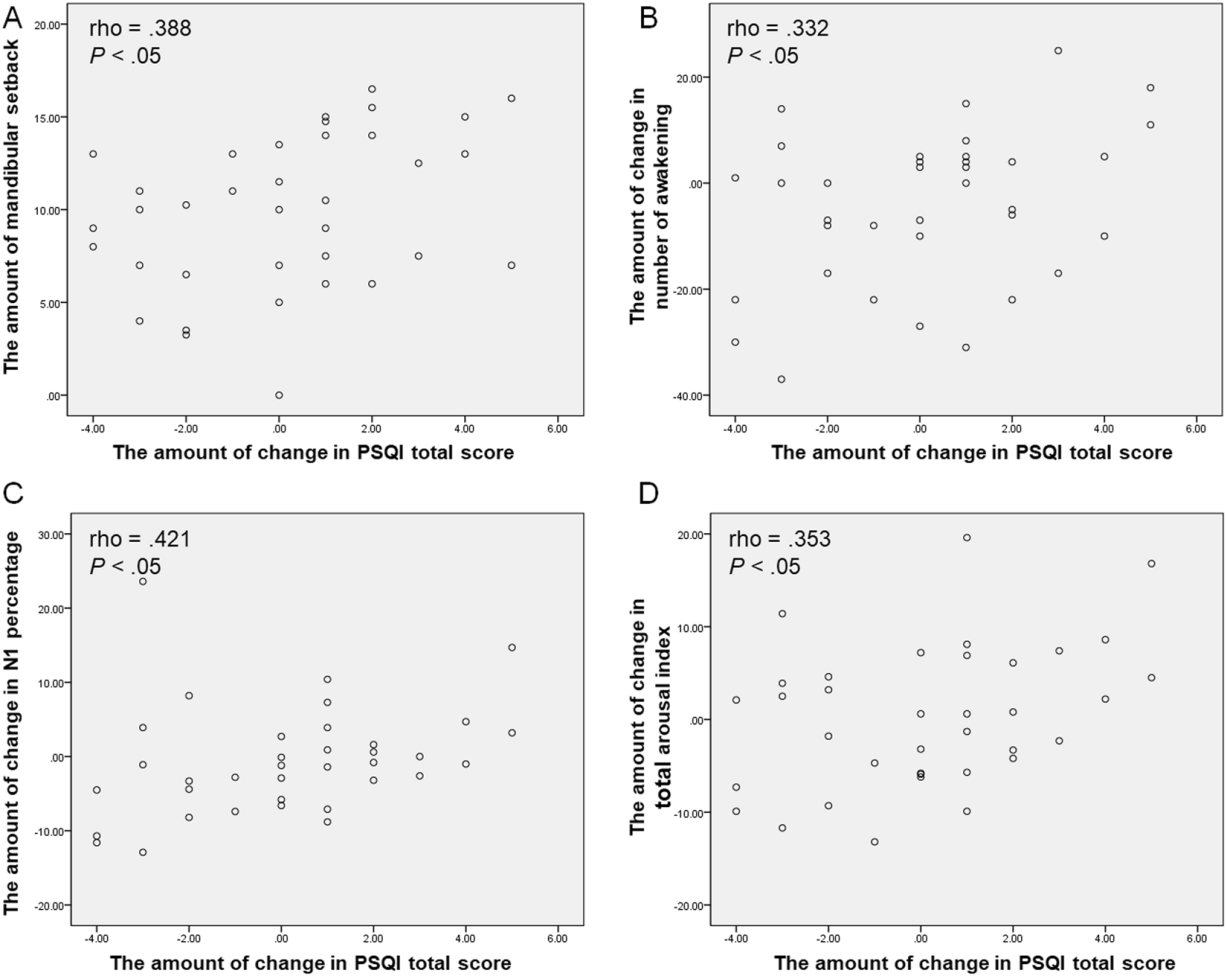
Scatter plots showing correlations between the PSQI and other variables. The amount of mandibular setback **(A)**, changes in number of awakening **(B)**, N1 percentage **(C)**, and total arousal index **(D)** showed positive correlation with the PSQI.

In terms of the subjective symptoms of patients, changes in the PSQI scores showed significant positive correlation with the amount of mandibular setback, changes in number of awakening, N1 percentage, and total arousal index (rho, 0.388, *P* < 0.05; rho, 0.332, *P* < 0.05; rho, 0.421, *P* < 0.05; rho, 0.353, *P* < 0.05; respectively) (Fig. 3). By contrast, changes in the ESS scores did not show correlation with other variables.

### Factors affecting the occurrence of OSA following MSS

The univariate logistic regression and multiple stepwise logistic regression of the occurrence of OSA versus preoperative PSG variables and the amounts of mandibular setback are shown in Table 3. The risk of occurrence of OSA increased significantly with increases in BMI and relative snoring time (both *P* < 0.05; Table 3), but decreased with increases in lowest oxygen saturation (*P* < 0.01; Table 3). Multiple stepwise logistic regression with additional insertion of the amounts of mandibular setback as an independent variable selected preoperative relative snoring time, and lowest oxygen saturation.

**Table 3:** Univariate and multivariate logistic regression of the occurrence of obstructive sleep apnea (OSA) versus preoperative and demographic variables. ^a^Statistical analysis was carried out using univariate logistic regression (the occurrence of OSA vs. preoperative and demographic variables).

Preoperative Variables	Univariate Analysis			Multivariate Logistic Regression		
	OR	95% CI	*P* Value^a^	OR	95% CI	*P* Value^b^
Relative snoring time (%)	1.062	1.012–1.115	0.015^*^	1.096	1.017–1.182	0.017^*^
Lowest oxygen saturation (%)	0.486	0.299–0.788	0.003^*^	0.427	0.235–0.775	0.005^*^
BMI (kg/m2)^†^	1.456	1.084–1.957	0.013^*^	none	none	0.617
Female gender^†^	0.119	0.014–1.042	0.054	none	none	0.855
The amount of mandibular setback (mm)^†^	1.126	0.923–1.375	0.242	none	none	0.438
Total sleep time (minutes)^†^	1.010	0.995–1.025	0.202	none	none	0.640
Sleep efficiency (%)^†^	1.067	0.980–1.162	0.133	none	none	0.929
Wake after sleep onset (minutes)^†^	0.985	0.965–1.006	0.164	none	none	0.983
Total RERA index (number/h)^†^	1.121	0.934–1.346	0.221	none	none	0.981

^b^Statistical analysis was carried out using multivariate logistic regression (the occurrence of OSA vs. preoperative and demographic variables). ^*^P < 0.05. ^†^Variables not significant in multivariate analysis (those exempted from the equation).

## Discussion

In this prospective study using overnight full PSG and 3D analysis with cone beam computed tomography (CBCT), MSS with or without maxillary surgical movement for skeletal class III patients resulted in significant changes in the sleep parameters of PSG, and volumes of PAS. Symptomatic sleep parameters including the RDI, and total RERA index significantly increased (both *P* < 0.001; Table 1). Particularly, even though BMI showed significant decrease after surgery (*P* < 0.001; Table 1), there were significant increases in the RDI, and total RERA index. In all types of PAS except for the nasopharyngeal airway, there were significant decreases in the volume, and the total volume of PAS also decreased significantly (all *P* < 0.001; Table 1). Several correlations between the sleep parameters in PSG and the amount of mandibular setback might indicate that the larger mandibular posterior movement is, the worse sleep quality becomes. Therefore, we suggest that MSS for skeletal class III patients could worsen sleep quality by increasing RDI, and total RERA index postoperatively. For this reason, it should be emphasized that there is a need to explain the possibility of deterioration of sleep quality after MSS in patients with skeletal class III patients, particularly in patients who require large amounts of mandibular setback.

It is well-known that the volume of total pharyngeal airway after MSS can be decreased. However, two main perspectives exist on the total volumetric change of the pharyngeal airway after MSS with maxillary advancement: significantly reduced^11–15^ and unchanged^16–21^. In terms of changes in the volume of regional PAS, the majority of articles have reported that the volume of the nasopharyngeal airway does not change significantly after two-jaw surgery^14,16,20,21^. In the present study, there were significant decreases in total pharyngeal airway volumes, whereas there was no significant change in the volume of the nasopharyngeal airway. These are consistent with previous studies.

Regarding the occurrence of OSA, although there is no evidence of postoperative OSA after MSS within the first 6 months^3^, 10 patients in this study showed postoperative OSA after MSS with or without maxillary surgery (MSS alone, 7; MSS with LF I, 3). Hasebe *et al*.^1^ reported two patients with postoperative OSA after MSS, and in those cases, the amounts of mandibular setback were large. Another study demonstrated that the patients who undergone mandibular setback greater than 5 mm had a higher incidence of mild to moderated OSA^7^. The mean of the amounts of mandibular setback in patients with postoperative OSA in the present study was also large (11.08 mm); however, one patient underwent MSS for only 4 mm setback. Based on this result, the occurrence of OSA might not always require large amount of mandibular setback. Because the individual host responses to altered biological circumstances such as narrowed upper airway space are different, customized treatment planning and surgery should be required to prevent the occurrence of iatrogenic OSA.

RERA is defined as a sequence of breathing events characterized by increasing respiratory effort, but which does not fulfill criteria for an apnea or hyponea^10^. On the basis of a report by American Academy of Sleep Medicine (AASM)^10^, it is suggested that RERAs and hypopneas share a similar pathophysiologic mechanism. Thus, RERA has been included in defining the OSA by using the same frequency of apneas and hypopneas since 1999. Nevertheless, attention has not been focused on RERA as a critical factor for OSA in the field of orthognathic surgery. To the best of our knowledge, there is no study which demonstrated changes in RERA after MSS or bimaxillary surgery. According to a position statement of AASM 2018^22^, as new studies increasingly uncover many negative outcomes associated with OSA, it is essential to accurately perform scoring all respiratory events, including those with arousal, in order to effectively diagnose and treat all patients with OSA. In addition, using PSG results that do not include arousal-based respiratory events of any form when scoring may lead to either lack of adequate diagnosis of OSA, misclassification of OSA severity, or misidentification of another sleep disorder or medical disorder^22^. In the present study, it was possible to score all respiratory events such as RERA, and consequently, it was observed that total RERA index significantly increased after MSS with or without LF I. Therefore, the authors insist that overnight full PSG possible to measure all respiratory events should be performed to evaluate respiratory changes before and after surgery.

The ESS and PSQI are two of the most widely used self-report questionnaires. The ESS includes eight self-rated questions, and has the purpose to measure a patient’s habitual likelihood of falling asleep in daily life. An ESS score of more than 10 are regarded as the indication of significant sleepiness. The PSQI has 19 self-rated items for evaluation of subjective sleep quality. Higher PSQI scores indicate worse sleep quality. However, neither the ESS nor PSQI was designed for screening of a specific sleep disorder. In addition, Nishiyama *et al*.^23^ demonstrated that because ESS and PSQI scores were more affected by psychological symptoms than PSG indices, they should not be used as screening or diagnostic methods for sleep disorders defined by PSG. In the present study, neither the ESS nor PSQI showed significant changes in their scores after surgery, even though postoperative OSA took place in some patients. These findings are consistent with previous studies showing inconsistency in the results of ESS and PSQI with objective sleep measures such as PSG indices. Nevertheless, the ESS and PSQI are warranted before and after surgery, because they can evaluate subjective symptoms. Regarding subjective symptoms, the most noteworthy finding in this study was that the RDI and RERA index could increase after MSS, although subjective symptoms did not show significant changes. Therefore, it is recommended that preoperative and postoperative evaluation by PSG is inevitable to detect these silent changes in patients who require MSS.

The present study differs from previous studies, in that it was a prospective study using preoperative and postoperative overnight full PSG and CBCT data to find out changes in the sleep parameters and volumes of PAS after MSS. In addition, the study also investigated the correlations among PSG, CBCT, and other demographic data and the possibility of the presence of factors affecting the occurrence of OSA. Although Gokce *et al*.^24^ also used overnight PSG and computed tomography and reported the changes in PAS and OSA measurements after bimaxillary orthognathic surgery in patients with class III malocclusion, our results differ from their findings. Their study demonstrated that bimaxillary orthognathic surgery caused an increase in the total airway volume and improvement in PSG parameters, and some correlations were reported between computed tomography and PSG parameter measurements. However, they focused on the combined effects of mandibular setback and maxillary advancement and did not present the detailed parameters in overnight full PSG such as the RDI, total RERA index, relative snoring time, and lowest oxygen saturation. Consequently, their results could not reveal distinct correlations between the sleep parameters in PSG and changes in the computed tomography data and could not identify risk factors affecting the occurrence of OSA. Tepecik *et al*.^25^ conducted another study that used CBCT data and overnight full PSG to examine the effects of bimaxillary orthognathic surgery on PAS and respiratory function during sleep. Despite presenting RDI, they did not show detailed PSG data and did not identify significant correlations between the PSG parameters and volumetric and area parameters in CBCT. The present study, in addition to the aforementioned distinctions, was performed on more than twice as many cases as those in studies conducted by Gokce *et al*. and Tepecik *et al*. Therefore, our report is the largest prospective study to date, comprising 50 patients with skeletal class III malocclusion, and using overnight full PSG for evaluation of sleep-related variables after MSS with or without maxillary surgical movement. Moreover, the present study has the originality, because it supports the finding for the first time that increases in RERA could result from MSS.

Although this is the first study to report significant changes in the RERA index by using preoperative and postoperative overnight full PSG examination prospectively following MSS in patients with skeletal class III patients, the present study has some limitations. This study could not evaluate the long-term effects of MSS. Postoperative PSG examination and CBCT taking were conducted 3 months after surgery, and the 3D volume analyses on PAS were performed by using the CBCT data. Van der Vlis *et al*.^26^ quantified the changes in postoperative swelling after orthognathic surgery using serial 3D photographs, and demonstrated that approximately 20% of the initial edema remained after 3 months, and significant decreases in soft tissue swelling still occurred between postoperative 6–12 months. Therefore, residual postoperative swelling in the soft tissue of the pharyngeal airway might affect the results of postoperative PSG and PAS volume measurements. In the future, it is necessary to investigate the long-term effects of MSS for skeletal class III patients on sleep quality or the occurrence of OSA by performing serial overnight full PSG whenever possible.

In conclusion, MSS with or without maxillary movement for skeletal class III patients could worsen sleep quality by increasing the sleep parameters in PSG. Although subjective symptoms may not show significant changes, objective sleep quality in PSG might decrease after MSS. It should be stressed that there is a need to explain the possibility of deterioration of sleep quality after MSS in patients with skeletal class III patients, particularly in patients requiring large amounts of mandibular setback, and overnight full PSG should be performed preoperatively and postoperatively for evaluation of the occurrence of OSA following MSS. Further studies are required to identify the long-term effects of MSS on sleep quality.

## Methods

### Patients

A total of 50 patients were included in this prospective study. All patients were selected preoperatively by following inclusion and exclusion criteria.

Inclusion criteria:

1. Medically healthy patients over 15 years of age.
2. Patients with skeletal and dental class III malocclusion showing anterior overjet of 0 or less, and ANB angle of 0 or less.
3. Patients requiring MSS alone or combined mandibular setback and Le Fort I osteotomy (LF I) to correct facial profile and malocclusion.
4. Patients without any respiratory diseases with possibility to influence the results of PSG.

Exclusion criteria:

1. Craniofacial anomaly patients.
2. Patients with a previous history of trauma to the face, and post-traumatic deformity.
3. Patients with severe facial asymmetry showing more than 5 mm of lower dental midline deviation from the upper dental midline.
4. Patients with endocrine diseases such as diabetes mellitus and hyperparathyroidism.

The study was conducted from December 2014 to May 2018. All of the enrolled patients provided signed informed consent forms before enrollment and had undergone orthognathic surgery consisting of bilateral sagittal split osteotomy (BSSO) alone or BSSO with concomitant LF I. Surgical procedures were performed by a single surgeon (S.I.S.) at Ajou University Hospital. The amounts of maxillary advancement and posterior impaction ranged from 0 to 5 mm (mean, 1.57 mm), and from 2.5 to 6 mm (mean, 4.61 mm), respectively. Genioplasty was additionally performed to improve the facial profile immediately after surgery if needed. The range of genial advancement was −4 to 4 mm (mean, −0.36 mm). Skeletal rigid fixation with titanium miniplates was applied, and all patients underwent maxillomandibular fixation for postoperative 2 weeks. The patient demographics are presented in Table 4. The Institutional Review Board of Ajou University Hospital approved this study in accordance with the regulations and guidelines (AJIRB-MED-MDB-14-397).

**Table 4:** Patient demographics.

Demographic	Value
**Age (year)**	
Mean ± SD	21.86 ± 4.55
Range	15–40
Sex	
Male	29
Female	21
**Amount of setback (mm)**	
Mean ± SD	9.55 ± 4.14
Range	2–16.5
Performed type of surgery	
BSSO (male: female)	36 (22: 14)
BSSO + LF I (male: female)	14 (7: 7)
**Performance of genioplasty**	
Performed	11
Not performed	39
**Existence of OSA**	
Preoperative OSA (mild: moderate: severe)	2 (1: 1: 0)
Postoperative OSA (mild: moderate: severe)	10 (8: 2: 0)

### Overnight PSG

All participants underwent standard full PSG (Embla® N7000; Natus Medical Inc., San Carlos, CA, USA) at the T0 and T1 in Ajou University Hospital. The standard PSG examination consisted of a six-channel electroencephalogram, two-channel electrooculogram, submental and leg electromyogram, electrocardiogram, and airflow (thermistor and pressure transducer), respiratory effort (chest and abdominal movement), oxygen saturation, snoring, and body position sensors. A qualified sleep technician manually scored all PSG data according to the AASM 2012 scoring rules^27^. All patients were requested not to drink any alcoholic or caffeinated beverages 24 hours before the sleep study.

Self-report questionnaires including the ESS and PSQI were administered to all enrolled patients shortly before recording at T0. However, postoperative questionnaires were completed only by patients who wanted to fill in the information. Consequently, no postoperative questionnaires were completed in 14 cases.

### CBCT image acquisition and volumetric measurement of airway space

**Figure 4:**
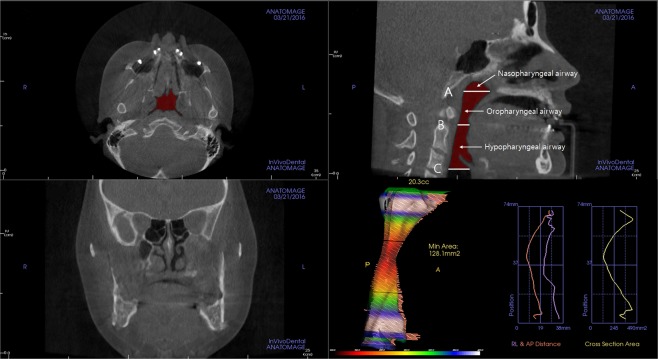
Three-dimensional measurement of the volume of PAS and reference planes and the division of pharyngeal airway space for volumetric measurement. Cone beam computed tomography data were imported to the program for visualizing pharyngeal airway space (PAS) and measuring the volume of PAS. Reference planes were set as follow: (**A**), posterior nasal spine (PNS) plane (axial plane passing through the PNS); (**B**), uvula plane (axial plane passing through the tip of the uvula); (**C**), epiglottis plane (axial plane passing through the base of the epiglottis). The pharyngeal airway was divided into three spaces based on the reference planes: the nasopharyngeal airway (between the highest point of the upper airway and the PNS plane), the oropharyngeal airway (between the PNS plane and the uvula plane), and the hypopharyngeal airway (between the uvula plane and the epiglottis plane).

CBCT scans were taken at T0 and T1 stages using the DINNOVA 3 dental-imaging system (HDX WILL Corp., Seoul, Republic of Korea) in 85 kV and 8 mA (scanning time, 25 seconds; field of view, 20 × 19 cm; slice, 0.3 mm). All patients were seated in an upright sitting position with a natural head position, and were asked to bite a positioning stick with the anterior teeth along the path of centric occlusion. The lips and tongue were kept in rest position during the scan. The head position was standardized by a laser light of the scan machine projected both onto the glabella and philtrum longitudinally, and onto the transverse line between the lateral eye canthus horizontally. The scanned data were converted to the Digital Imaging and Communications in Medicine format, and imported to the Invivo 5.1 (Anatomage, San Jose, CA, USA) program for visualization and measurements of PAS (Fig. 4). Volumetric measurements of the airway were performed after establishment of horizontal reference planes to divide PAS into three spaces: nasopharyngeal space, oropharyngeal space, and hypopharyngeal space. Landmarks and references were identified as shown in Fig. 4. Threshold values for measurements were set at a range of −1024 to −400 Hounsfield units to remove artifacts and to clarify each airway space. All measurements were performed by a single examiner (S.W.O.), and all measurements was repeated after a 2-week interval by the same examiner. Intraobserver reliability calculated by Pearson correlation coefficient showed excellent agreement (r, 0.999, *P* < 0.001). The total volume of PAS was calculated by summing the measured volumes of the nasopharyngeal airway, oropharyngeal airway, and hypopharyngeal airway.

### Statistical analysis

Descriptive statistics were used to calculate the mean, and standard deviation of the variables obtained from the patient data. The Shapiro-Wilk test and Kolmogorov-Smirnov test were performed to evaluate data normality. The differences between T0 and T1 were determined using Wilcoxon-signed rank test, and Bonferroni adjustment was applied for multiple comparison correction. Spearman’s correlation analysis was used to assess correlations among the variables. After 14 patients who did not complete postoperative questionnaire were excluded, correlations between the subjective symptoms of patients and other variables were analyzed by performing Spearman’s correlation analysis. The influence of preoperative variables on the occurrence of OSA was analyzed using univariate and stepwise multiple logistic regression after 2 patients with preoperative OSA were excluded. *P* values of less than 0.05 were considered statistically significant. All statistical analyses were conducted by using SPSS 21.0 (SPSS Inc, Chicago, IL, USA).

### Ethical approval and informed consent

This prospective clinical study was approved by The Institutional Review Board of Ajou University Hospital, and performed in accordance with the regulations and guidelines (AJIRB-MED-MDB-14-397). Informed consent was obtained from each participants. For participants under the age of 18 years, informed consent was obtained not only from a parent and/or legal guardian, but also from the participants themselves.

## Data Availability

The datasets generated during and/or analyzed during the current study are available from the corresponding author on reasonable request.
